# Personalized Game-Based Content and Performance: A Pilot Study on a Digital Intervention for Children with ADHD

**DOI:** 10.3390/bioengineering11121277

**Published:** 2024-12-16

**Authors:** Seon-Chil Kim, Jeong-Heon Song, Na-Yeong Kong

**Affiliations:** 1 Department of Biomedical Engineering, School of Medicine, Keimyung University, 1095 Dalgubeol-daero, Daegu 42601, Republic of Korea; 2 AI-Based Neurodevelopmental Diseases Digital Therapeutics Group, Korea Brain Research Institute (KBRI), 61, Cheomdan-ro, Daegu 41062, Republic of Korea; jhsong@kbri.re.kr; 3 Department of Psychiatry, School of Medicine, Keimyung University, Daegu 42601, Republic of Korea; kp8937@kmu.ac.kr

**Keywords:** digital intervention therapy, engagement, difficulty, content, motivation

## Abstract

Mobile-based digital interventions for children with attention-deficit hyperactivity disorder (ADHD) have been developed to alleviate their symptoms. When developing mobile game-based digital interventions for ADHD treatment, it is important to research how the emotional responses of the target audience members—based on flashy visuals or difficulty adjustments to motivate the user—affect their content manipulation ability. This study performed a correlation analysis to examine the impact of perceived difficulty and enjoyment (interest) on the performance of children diagnosed with ADHD while engaging in game-based digital content. Statistically significant differences were observed in the following variables based on the enjoyment level: correct rate (*p* = 0.0040), decision time (*p* = 0.0302), difficulty (*p* < 0.0001), and touch time (*p* = 0.0249). Considering difficulty level, statistically significant differences were observed for correct rate (*p* = 0.0011), decision time (*p* = 0.0158), and difficulty (*p* < 0.0001). Correlation analysis between the variables correct rate, decision time, difficulty, touch, time limit, and touch time based on enjoyment and difficulty did not reveal significant correlations. Therefore, for children with ADHD, digital interventions should focus on the therapeutic goals rather than on flashy visuals or difficulty adjustments aimed at enhancing interest. Based on these results, further research exploring how psychological states affect performance regarding digital content is necessary.

## 1. Introduction

Attention-deficit hyperactivity disorder (ADHD) is one of the most common childhood mental health disorders and is typically treated with a combination of medication and behavioral therapy [1,2,3]. However, concerns about medication adherence and side effects make it common practice to use both treatment methods together. Recent advancements in digital technologies have introduced various non-pharmacological behavioral therapies, with digital intervention emerging as a prominent approach [4]. This type of therapy applies game-based digital content to the daily lives of children with ADHD and has shown positive results [5,6]. Studies on digital intervention have demonstrated the positive effects of game-based content in children with ADHD, contributing to the diagnosis and treatment of this disorder [7,8]. Mobile-based digital games have proven highly effective for children aged 8–12 years [9], with game-based digital content reported to have a positive impact on motivation and treatment goals [10,11]. Children with ADHD typically experience boredom with treatment and evaluation tools; therefore, maintaining sustained motivation and interest is critical for effective therapy [12].

In video game-based content, the difficulty level is adjusted step-by-step according to the child’s learning ability; therefore, the results of the game level during the treatment process are reflected in the therapeutic outcomes [13,14]. Additionally, digital content is used in a similar form for improving cognitive function, such as in the case of dementia or cognitive impairment [15,16]. This approach is actively used to develop interventions aimed at improving processing speed, working memory, and attention [17,18]. In the case of such game-based content, younger participants are more likely to be directly influenced by the structure of the content. Elements such as screen design, game storyline, and the speed of visual transitions may vary in their impact depending on the participant’s age. In behavioral therapy using game-based digital content for individuals with ADHD, participants engage directly with the content while medical professionals and caregivers monitor it, which limits direct intervention [19,20]. As such, it is difficult to individually tailor the game and content to a participant’s cognitive situation; however, the difficulty levels are customized based on the results of their performance.

Interventions using game-based digital content could be influenced by emotional regulation issues or a decline in self-esteem during the gaming process, which may affect treatment outcomes for participants with ADHD [21,22]. These concerns can also pose challenges for developers who create game-based digital content for therapeutic purposes, as well as healthcare professionals who recommend or prescribe these interventions [23]. The enjoyment and difficulty experienced by the participant while engaging with the content may impact the therapeutic effect, depending on how well the participant understands the developer’s intentions [24]. Hence, communication between physicians and developers regarding content design is crucial. Research remains limited in terms of how the difficulty level or the use of engaging with characters in game-based digital content affects accuracy rates in children with ADHD [25]. Furthermore, within the same level of content, the difference in difficulty based on the participants’ understanding of the content has not yet been sufficiently investigated through case studies and research reports [26].

The present study conducted a correlational analysis to examine the impact of emotional responses, such as level of difficulty and engagement, on the performance of children with ADHD when taking part in mobile game-based digital interventions. The elaborate visual design of the content, which includes various elements such as color, shape, degree of change, and social popularity, is intended to stimulate the motivation of the children and play an active role in the therapeutic process, thereby serving as an object of interest. After the children interacted with the developed content, their subjective evaluations of difficulty and engagement were quantitatively assessed to determine the effects on content performance. The findings provide insight into the psychological changes experienced during digital intervention therapy and highlight the importance of understanding these effects with regard to task performance. The results serve as a guide for the future development of content for game-based digital interventions, particularly regarding how components such as screen design and characters’ features should be expressed. In addition, this study aims to minimize the gap between developers creating content and healthcare professionals prescribing or recommending its use.

## 2. Materials and Methods

### 2.1. Participants

This study was conducted at the Department of Psychiatry at Keimyung University, specifically at Dongsan Hospital, from December 2022 to March 2023. Ethical requirements for research on human participants were considered, and approval from the Institutional Review Board (IRB) of Keimyung University, Dongsan Hospital (IRB No. 2022-10-019, CRIS Registration No. KCT0009862) was obtained before data collection and evaluation. The informed consent form and explanation included information on the study’s purpose, participation procedures, potential risks and benefits, confidentiality, and assurance that the collected data would not be used except for the purpose of the study. Data collection and experimentation began after written consent was obtained from the participants and their guardians. Participants were selected based on a comprehensive assessment, including clinical interviews with a psychiatrist and psychological tests, such as attention and intelligence assessments. The data were stored and securely protected in accordance with the Bioethics and Safety Act.

This study involved 16 children aged 6–13 years who were diagnosed with ADHD according to the diagnostic criteria of the *Diagnostic and Statistical Manual of Mental Disorders, Fifth Edition* [27]. The inclusion criteria were as follows: voluntary written consent from the legal guardian for participation in the clinical trial; agreement from the participants themselves; ability to comply with the clinical trial evaluation and instructions from the principal investigator; and normal intellectual functioning based on the Korean version of the Wechsler Intelligence Scale for Children [28].

**Figure 1:**
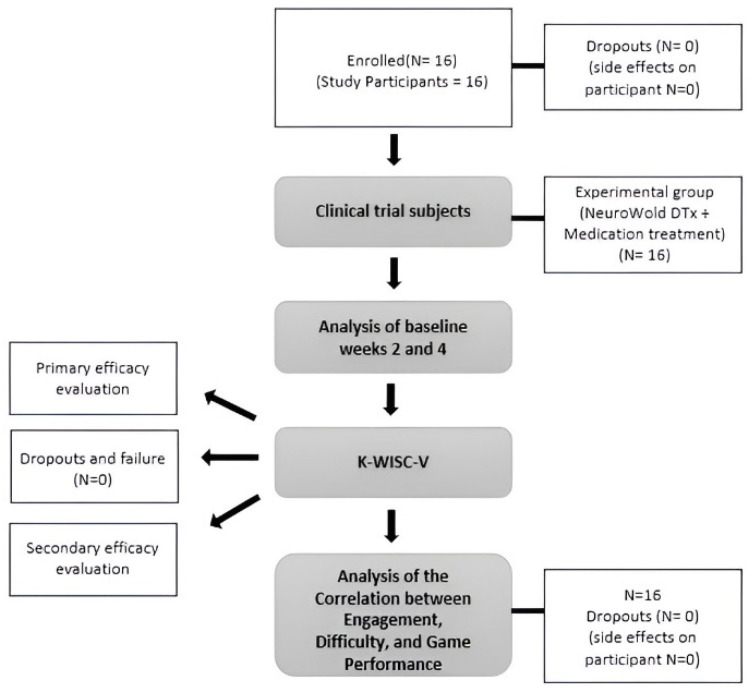
Flow diagram of the participants.

The exclusion criteria for participation were as follows: individuals with other disorders or diseases in addition to ADHD (e.g., post-traumatic stress disorder, psychosis, severe obsessive–compulsive disorder, or severe depression); those with conditions that could affect the use of experimental products (e.g., physical deformities of the hands or arms, or prosthetic limbs); individuals with a history of drug abuse or dependence confirmed or suspected within the past six months; those who had participated in other ADHD-related studies within 90 days of screening; individuals with color blindness; family members who were enrolled or participating in the same study; and those who were deemed unsuitable for participation. Children who met any of these criteria were excluded. Although no direct adverse effects were expected in the participating children, frustration, headaches, and dizziness may have occurred as a result of using the application owing to the digital device’s characteristics. To mitigate this, the device usage time was limited to 30 min or less using an in-program time-monitoring feature [29]. The overall structure of the study is shown in Figure 1.

### 2.2. Experimental Methods

**Figure 2:**
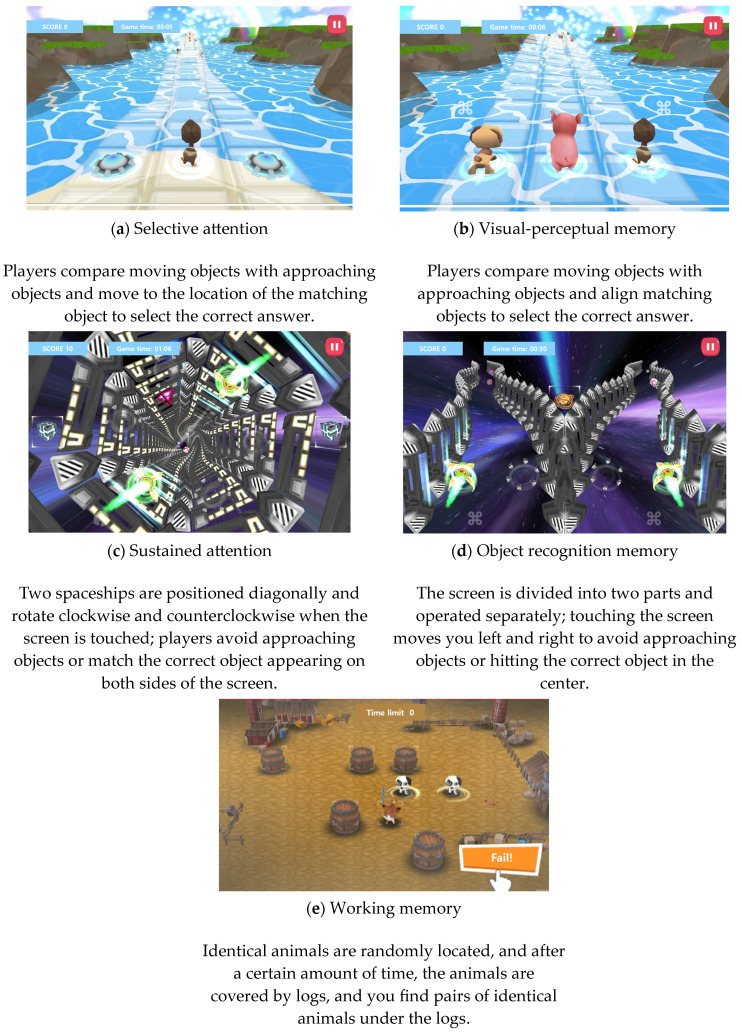
Screen with main content: (**a**) Selective attention; (**b**) visual-perceptual memory; (**c**) sustained attention; (**d**) object memory; (**e**) working memory.

This study used NewroWorld DTx, developed by Woori Soft Co., Ltd. (Deagu, Republic of Korea), as a digital therapeutic intervention tool. The participants utilized content structured by stage and grade level. The cognitive function therapy program was applied through a smart device (a tablet PC). It included attention training that involved working memory, the ability to remember and process a series of stimuli in order, and the ability to ignore surrounding distractor stimuli and respond to the necessary stimuli [30,31]. The content used in the experiment aligned with the study’s purpose, as it was structured as a game-based solution model featuring animal characters, as shown in Figure 2. The experimental group completed 20 sessions of the digital therapeutic intervention over four weeks, using it five days a week and engaging with the content for 25 min each day. Starting on the first day of participation, two surveys were administered after each week of content execution. They asked whether the content was difficult or easy and whether the participant had engaged with it. Therefore, five surveys were conducted (on days 1, 5, 10, 15, and 20) and data were collected.

### 2.3. Analysis Methods

A correlation analysis was performed to examine the links between the correct rate, decision-making time, difficulty level, the number of times the screen is touched, time limit, and touch time, as well as the engagement and difficulty levels of the content. Statistical analysis was performed on data collected from participants with treatment results using as-is data without any substitutions for missing values. All statistical analyses were performed using SAS Software (version 9.4, SAS Institute, Cary, NC, USA). Descriptive statistics included the number of participants, mean, standard deviation, and median, minimum, and maximum values. Statistical significance for the difference between groups was determined using an independent two-sample t-test to establish if normality was satisfied when comparing the two groups. The Wilcoxon rank sum test was employed to ascertain if normality was not satisfied, and analysis of variance (ANOVA) and post hoc tests were performed when comparing three or more groups [32,33,34]. The statistical significance of the correlation between two variables was tested using Pearson’s correlation, and all statistical tests were performed with two-tailed tests at a significance level of 0.05 [35]. Numerical values (including decimal places, such as the average, standard deviation, and percentage) were expressed by rounding from the third to the second decimal place. Simultaneously, correlation coefficients, regression coefficients, and *p*-values were presented to four decimal places; if the *p*-value was less than 0.0001, it was represented as <0.0001.

## 3. Results

### 3.1. Participant Information

The characteristics of the participants are shown in Table 1. There were fewer female than male participants, and none of them had comorbid conditions.

### 3.2. Correlation Analysis

In Table 2 and Table 3, an attempt was made to visually confirm the correlation between enjoyment and difficulty level for each variable (correct rate, decision-making time, difficulty level, touch, time limit, and touch time). However, no significant correlations were found. Hence, engagement and difficulty level in the content have little correlation with each variable.

In Table 4 and Table 5, descriptive statistics (the mean, standard deviation, and median, minimum, and maximum values) for the total number of games performed by the participants are presented based on the categories of “engagement” and “difficulty”, along with the *p*-values from the ANOVA. Statistically significant differences were observed for the correct rate (*p* = 0.0040), decision-making time (*p* = 0.0302), difficulty level (*p* < 0.0001), and touch time (*p* = 0.0249) according to the level of engagement, as well as for the correct rate (*p* = 0.0011), decision-making time (*p* = 0.0158), and difficulty level (*p* < 0.0001) according to the difficulty level.

Therefore, regarding users’ subjective evaluation of engagement with the content, as the level of engagement increased, the correct rate increased, decision-making time decreased, the difficulty level increased, and touch time decreased. Additionally, according to the evaluation based on the difficulty level encountered in the content, as users judged the difficulty level to be easier, the correct rate increased, decision-making time decreased, and the difficulty level increased.

As shown in Table 4 and Table 5, statistical significance was identified between the variables (correct rate, decision-making time, difficulty level, touch, time limit, and touch time) and the levels of engagement and difficulty. There were some points where the direction of interpretation was unclear, so some of the categories within engagement and difficulty level were integrated and reanalyzed, as shown by the results in Table 6 and Table 7. Previously, results were divided into five categories: engagement and difficulty level. The additional analysis simplified these into two categories: games perceived as fun versus not fun, and games perceived as easy versus not easy. The “engaging” category included the previous “outstanding” and “engaging” levels, while the “easy” category included the previous “extremely easy” and “easy” levels. In this analysis, significant differences were observed for difficulty level (*p* = 0.0004) and touch time (*p* = 0.0135) based on the level of engagement. Games perceived as more engaging were associated with higher difficulty scores and shorter touch times. Regarding difficulty levels, significant differences were observed in the variables of difficulty level (*p* = 0.0003) and touch (*p* = 0.0132); games perceived as easier were associated with higher values for difficulty level and touch.

## 4. Discussion

Video game-based content—widely used for digital interventions targeting children—can be used as a learning tool, helping children maintain accessibility and focus during performance and stimulating various senses [36,37,38].

When such digital content is utilized as an adjunctive therapy for children with ADHD, it is typically designed with content groups and level-based modules. Consistency in content is considered a critical factor, as it primarily focuses on symptom assessment and presenting therapeutic outcomes [39]. The consistency of content during performance refers to the game’s difficulty level in the same-level tier of the game’s format. The outcomes for each difficulty level are related to the results of treatment and training for individuals with ADHD, and the evaluator analyzes these results [40]. As such, the developed program has different content based on difficulty level, and the users perceive such difficulty levels as they progress through the stages and take part in the training. Hence, users may experience perceived difficulty at each level and within the same content differently. However, from the developer’s perspective, maintaining consistency in the level of difficulty is a challenging task, as users engage with various types of content to stay focused on the program. Even at the same stage, differences in difficulty level can create new motivational elements for a program [41].

In this study, the degree to which users perceived the difficulty of the content provided by the developer varied, and the evaluation of the difficulty in the results corresponded to individual factors. As such, the definition of difficulty refers to the comparative difficulty between forms of content perceived by users within the same level. Although the participants experienced some difficulty performing the task, it did not significantly affect the results. Analyzing the outcomes, the difficulty of the content perceived by the user during performance is an individual capacity. This does not significantly affect digital intervention therapy as it is a natural part of performance. These results provide a theoretical basis for developers to present various problems rather than repeating the same content to maintain consistency in terms of difficulty. Therefore, developing games with stories with a clear beginning, middle, and end—rather than focusing solely on screen-based content—could be feasible [42,43]. The second finding, regarding user perceptions of engagement and interest in the content, is also a personal outcome similar to difficulty. This result indicates that even if a developer creates visually elaborate content, it does not significantly improve the accuracy rate during performance. Although there may be individual differences in the perception of engagement and interest, these did not affect the accuracy rate, determining the therapeutic effect.

Such findings indicate that it is important to establish a clear planning intention for the entire content development process and to set specific training goals for the target audience. The content used in digital intervention therapy has a direct or indirect temporary impact on treating a condition, and its purpose is to improve the environment through repetitive performance [44]. To achieve this, it is essential to use content that aligns with the planned intention. In this case, repetitive screens for consistency in difficulty level and excessive screen designs focused on engagement and interest would not be helpful. Maintaining consistency through content that requires judgment and memory in the same functional context is more appropriate [45].

For digital intervention therapy content designed for children with ADHD, accessibility and sustainability are crucial elements to achieve positive effects. To ensure success in these areas, the content should incorporate engaging characters and competitive elements during the performance process. These features cannot be solely based on simple accessibility but must also take into account the psychological aspects of the target children [46].

The correlations linking the survey variables of difficulty level and engagement with the variables of correct rate, decision-making time, difficulty level, touch, time limit, and touch time were analyzed to derive quantitative results. There was almost no correlation between the psychological factors of difficulty level and engagement (as perceived by the performer) and other variables. This suggests that when developing the content used in a digital intervention, it is important for the content to align with the target audience’s therapeutic goals. Furthermore, for the content employed in digital intervention therapy to expand its use as an adjunctive effect in future digital therapeutics, a phased testing process aligned with the planning intention and therapeutic goals will be necessary throughout the development phase. Additionally, given that environmental factors such as game difficulty and characters (which differ for each individual in the problem-solving modules of game-based content) have little impact on performance outcomes, if developers create content tailored to digital intervention therapy, the results will ultimately maintain consistency.

The design of content components impacts both children with ADHD and clinicians when developing digital therapeutic content. For children with ADHD, story-based content with active motivation can positively affect repetitive learning and training. For clinicians, game-based content offers the advantage of serving as a conversational topic to engage with patients. Therefore, from the developer’s perspective, the design of the content is crucial.

This study has several limitations. First, it was a small-scale study conducted over four weeks involving only 16 patients with ADHD. Although the statistical effects of ADHD symptoms were confirmed in the 16 participants, we aim to explore the long-term effectiveness in future large-scale studies. Second, this study included more male than female participants. According to research on ADHD and neurodevelopmental disorders among children aged 6–13 years, the male-to-female ratio of ADHD prevalence is approximately 2:1, with a higher incidence of ADHD in boys because of biological and sociocultural factors [47]. Third, the users’ perceived difficulty and engagement with the content were highly subjective, and an experimental method that did not replicate the content using similar materials for the control group was chosen. This aspect has the drawback of being unable to respond actively to changes in a user’s psychological state. In addition, owing to the nature of research involving digital media, there are concerns regarding issues such as device addiction and misuse [48]. To prevent the negative effects of prolonged engagement, this study limited the daily usage time to 30 min or less, which helped increase the satisfaction of the participating children’s parents.

In future large-scale studies, we aim to analyze the necessary components in the development process of digital content used in digital interventions and evaluate the efficacy of the content for children with ADHD, as well as the therapeutic effects based on the participants’ psychological state. In doing so, we aim to provide developers of digital therapeutics with guidelines for content types based on examples of components. Additionally, this study evaluates content from the perspective of developers who will create digital content in the future. As such, it was understood that the fundamental goals— such as the planning intention and therapeutic objectives—are more important than the design or layout of the supplementary content.

## 5. Conclusions

This study analyzes the impact of perceived difficulty and fun elements on performance when children diagnosed with ADHD engage in game-based digital content for digital intervention therapy. A correlation analysis was conducted to examine the relationships between the levels of fun and difficulty and performance indicators such as correct rate, decision time, difficulty, touch, time limit, and touch time. The results showed no statistically significant correlations.

These findings can contribute to the development of new guidelines for the design of digital content aimed at mental health treatment and training. Specifically, this study highlights the importance of aligning the content design with therapeutic objectives, rather than focusing on overly elaborate characters or difficulty levels adjusted through repetitive consistency. This evidence underscores the significance of therapeutic alignment in the development process.

Future digital intervention therapy content should prioritize therapeutic efficacy and symptom improvement over purely technical factors such as accessibility, age-specific suitability, and program compatibility. Developing story-based content that considers direct adaptation to therapeutic goals could foster active engagement among target children.

## Figures and Tables

**Table 1:** Information on the participating children.

		Experimental Group (N = 16)		Accompanying Symptoms
		N	%	
**Gender**	**Male**	13	81.3	None
	**Female**	3	18.7	
		Mean	SD	
**Age (years)**		8.63	1.86	None

**Table 2:** Results of correlation analysis by level of engagement.

Correlation Analysis (vs. Engagement Level)	Correct Rate	Decision-Making Time	Difficulty Level	Touch	Time Limit	Touch Time
**Pearson’s correlation coefficient (r)**	0.1151	−0.1682	0.2059	−0.0024	−0.0261	−0.2863
**(95% CI)**	(0.0268, 0.2016)	(−0.2774, −0.0546)	(0.1195, 0.2892)	(−0.0910, 0.0862)	(−0.2234, 0.1732)	(−0.4603, −0.0910)

CI = confidence interval.

**Table 3:** Results of correlation analysis by difficulty level.

Correlation Analysis (vs. Difficulty Level)	Correct Rate	Decision-Making Time	Difficulty Level	Touch	Time Limit	Touch Time
**Pearson’s correlation coefficient (r)**	−0.0622	0.1400	−0.2033	−0.1020	0.0952	0.0698
**(95% CI)**	(−0.1499, 0.0266)	(0.0258, 0.2506)	(−0.2868, −0.1169)	(−0.1889, −0.0135)	(−0.1052, 0.2882)	(−0.1325, 0.2666)

CI = confidence interval.

**Table 4:** Level of fun: descriptive statistics and ANOVA results.

Subjective Evaluation	Output Results After Completing the Game						
Engagement		Correct Rate (%)	Decision-Making Time (s)	Difficulty Level	Touch (Frequency)	Time Limit (s)	Touch Time (s)
**Extremely unengaging**	E	75	43	75	75	22	8
	Mean (SD)	82.98 (17.38)	0.94 (0.61)	6.48 (3.64)	156.07 (113.83)	13.03 (4.46)	1.95 (0.43)
	Median	87.14	0.88	5.22	137.00	13.59	1.98
	Min, Max	0.00, 100.00	0.13, 2.29	1.00, 13.96	0.00, 719.00	0.00, 21.00	1.43, 2.57
**Not engaging**	E	21	12	21	21	8	1
	Mean (SD)	90.87 (6.27)	0.87 (0.59)	6.99 (4.30)	140.62 (93.21)	12.78 (0.97)	1.03 (-)
	Median	92.86	0.74	4.36	122.00	12.59	1.03
	Min, Max	74.51, 96.83	0.11, 2.01	1.11, 12.89	25.00, 471.00	11.39, 14.43	1.03, 1.03
**Moderately engaging**	E	133	76	133	133	29	27
	Mean (SD)	88.95 (12.43)	0.72 (0.74)	8.49 (4.26)	145.76 (95.71)	13.83 (2.83)	1.66 (0.50)
	Median	91.84	0.56	9.26	117.00	14.50	1.64
	Min, Max	22.22, 100.00	0.07, 4.49	1.00, 15.03	0.00, 801.00	6.00, 17.91	0.96, 2.55
**Engaging**	E	105	69	105	105	19	17
	Mean (SD)	89.17 (11.86)	0.79 (0.90)	9.00 (4.06)	141.55 (87.90)	12.28 (3.03)	1.51 (0.42)
	Median	92.45	0.66	9.91	119.00	11.86	1.53
	Min, Max	23.33, 100.00	0.06, 6.65	1.05, 14.89	44.00, 786.00	6.73, 17.19	1.00, 2.56
**Outstanding**	E	156	93	156	156	20	43
	Mean (SD)	88.51 (10.04)	0.55 (0.52)	9.04 (4.14)	153.60 (95.59)	13.05 (3.42)	1.46 (0.40)
	Median	91.51	0.39	10.04	125.50	12.18	1.34
	Min, Max	44.59, 100.00	0.09, 3.31	1.05, 15.59	34.00, 931.00	8.83, 20.84	0.93, 2.21
***p*** -value *		0.0040 ^(1)^	0.0302 ^(2)^	<0.0001 ^(3)^	0.7961	0.6220	0.0249 ^(4)^

E: Number of games. * ANOVA (Y = output results after the game/X = participant’s subjective evaluation [engagement]). (1) Independent *t*-test (extremely unengaging vs. moderately engaging: *p* = 0.0099; extremely unengaging vs. engaging: *p* = 0.0086; extremely unengaging vs. outstanding: *p* = 0.0121). (2) Independent *t*-test (extremely unengaging vs. outstanding: *p*-value = 0.0002). (3) Independent *t*-test (extremely unengaged vs. moderately engaged: *p* = 0.0007; extremely unengaged vs. engaged: *p* < 0.0001; extremely unengaged vs. outstanding: *p* < 0.0001). (4) Independent *t*-test (extremely unengaging vs. outstanding: *p*-value = 0.0029).

**Table 5:** Descriptive statistics and results of ANOVA by difficulty level.

Subjective Evaluation	Output Results After Completing the Game						
Difficulty		Correct Rate (%)	Decision-Making Time (s)	Difficulty Level	Touch (Frequency)	Time Limit (s)	Touch Time (s)
**Extremely easy**	E	131	90	131	131	13	26
	Mean (SD)	86.83 (14.15)	0.70 (0.83)	9.11 (4.30)	166.76 (124.45)	11.74 (3.02)	1.53 (0.50)
	Median	89.72	0.46	10.72	132.00	12.21	1.34
	Min, Max	20.83, 100.00	0.09, 6.65	1.00, 15.26	0.00, 801.00	4.50, 16.32	0.94, 2.57
**Easy**	E	69	46	69	69	12	11
	Mean (SD)	91.19 (7.49)	0.55 (0.43)	9.45 (3.98)	154.00 (76.03)	13.05 (3.24)	1.55 (0.48)
	Median	92.63	0.46	9.80	135.00	13.18	1.49
	Min, Max	68.57, 100.00	0.06, 1.77	2.29, 15.59	36.00, 457.00	6.73, 19.43	0.93, 2.21
**Moderate**	E	151	87	151	151	31	33
	Mean (SD)	89.45 (10.98)	0.65 (0.61)	8.55 (4.13)	139.29 (67.74)	13.79 (2.75)	1.54 (0.48)
	Median	92.16	0.48	9.91	121.00	13.93	1.44
	Min, Max	22.22, 100.00	0.07, 4.49	1.07, 15.03	18.00, 397.00	7.00, 18.94	0.96, 2.55
**Difficult**	E	65	27	65	65	23	15
	Mean (SD)	88.98 (10.47)	0.92 (0.85)	7.49 (4.02)	132.35 (67.53)	12.75 (2.80)	1.60 (0.43)
	Median	92.31	0.76	6.82	115.00	12.57	1.45
	Min, Max	46.67, 100.00	0.09, 3.05	1.05, 14.01	44.00, 453.00	6.00, 17.91	1.07, 2.56
**Extremely** **difficult**	E	74	43	74	74	19	11
	Mean (SD)	83.43 (15.88)	0.99 (0.68)	6.67 (3.78)	145.42 (123.55)	13.40 (4.70)	1.64 (0.37)
	Median	86.96	0.90	6.04	108.00	13.29	1.69
	Min, Max	0.00, 100.00	0.07, 3.31	1.00, 14.32	0.00, 931.00	0.00, 21.00	1.12, 2.22
***p*** -value *		0.0011 ^(1)^	0.0158 ^(2)^	<0.0001 ^(3)^	0.0859	0.4205	0.9612

E: Number of games. * ANOVA (Y = output results after the game/X = participant’s subjective evaluation [difficulty level]). (1) Independent *t*-test (easy vs. extremely difficult: *p* = 0.0003; moderate vs. extremely difficult: *p* = 0.0041). (2) Independent *t*-test (easy vs. extremely difficult: *p*-value = 0.0005). (3) Independent *t*-test (extremely easy vs. extremely difficult, *p* < 0.0001; easy vs. difficult, *p* = 0.0054; easy vs. extremely difficult, *p* = 0.0012).

**Table 6:** Descriptive statistics and results of the t-test by level of interest.

Subjective Evaluation	Output Results After Completing the Game						
Engagement		Correct Rate (%)	Decision- Making Time (s)	Difficulty Level	Touch (Frequency)	Time Limit (s)	Touch Time (s)
**Experienced** **engagement**	E	261	162	261	261	39	60
	Mean (SD)	88.77 (10.79)	0.65 (0.72)	9.02 (4.10)	148.75 (92.59)	12.67 (3.22)	1.47 (0.41)
	Median	91.84	0.42	10.01	124.00	12.14	1.35
	Min, Max	23.33, 100.00	0.06, 6.65	1.05, 15.59	34.00, 931.00	6.73, 20.84	0.93, 2.56
**Did not** **experience** **engagement**	E	229	131	229	229	59	36
	Mean (SD)	87.17 (14.13)	0.81 (0.69)	7.69 (4.16)	148.66 (101.49)	13.39 (3.37)	1.71 (0.50)
	Median	90.00	0.68	7.23	122.00	13.62	1.65
	Min, Max	0.00, 100.00	0.07, 4.49	1.00, 15.03	0.00, 801.00	0.00, 21.00	0.96, 2.57
*p*-value *		0.1641	0.0639	0.0004	0.9917	0.2971	0.0135

E: Number of games. * Independent two-sample *t*-test. Instances of the game being perceived as engaging are attributed to a combination of responses for the categories “engaging” and “outstanding”. Instances of the game being perceived as not engaging are attributed to a combination of responses for “moderate” and “extremely unengaging”.

**Table 7:** Descriptive statistics and results of *t*-test by difficulty level.

Subjective Evaluation	Output Results After Completing the Game						
Difficulty		Correct Rate (%)	Decision-Making Time (s)	Difficulty Level	Touch (Frequency)	Time Limit (s)	Touch Time (s)
**Felt it was easy**	E	200	136	200	200	25	37
	Mean (SD)	88.33 (12.42)	0.65 (0.72)	9.22 (4.19)	162.36 (110.14)	12.37 (3.13)	1.53 (0.48)
	Median	91.56	0.46	10.54	133.50	12.50	1.39
	Min, Max	20.83, 100.00	0.06, 6.65	1.00, 15.59	0.00, 801.00	4.50, 19.43	0.93, 2.57
**Did not feel it was easy**	E	290	157	290	290	73	59
	Mean (SD)	87.81 (12.54)	0.79 (0.69)	7.83 (4.09)	139.30 (85.25)	13.36 (3.36)	1.57 (0.44)
	Median	91.62	0.62	7.38	116.50	13.43	1.53
	Min, Max	0.00, 100.00	0.07, 4.49	1.00, 15.03	0.00, 931.00	0.00, 21.00	0.96, 2.56
*p*-value *		0.6496	0.0966	0.0003	0.0132	0.1981	0.6870

E: Number of games. * Independent two-sample *t*-test. Instances of the game being perceived as easy are attributed to a combination of responses for the categories “very easy” and “easy”. Instances of the game being perceived as not easy are attributed to a combination of responses for the categories “difficult” and “extremely difficult”.

## Data Availability

All data generated during this study are included in this manuscript.
